# Interdisciplinary collaboration across secondary and primary care to improve medication safety in the elderly (The IMMENSE study) – a randomized controlled trial

**DOI:** 10.1186/s12913-022-08648-1

**Published:** 2022-10-26

**Authors:** Jeanette Schultz Johansen, Kjell H. Halvorsen, Kristian Svendsen, Kjerstin Havnes, Eirin Guldsten Robinson, Hilde Ljones Wetting, Stine Haustreis, Lars Småbrekke, Elena Kamycheva, Beate Hennie Garcia

**Affiliations:** 1 Department of Pharmacy, Faculty of Health Sciences, UiT the Arctic University of Norway, Tromsø, Norway; 2 Surgery, Cancer and Women’s Health Clinic, The University Hospital of North Norway, Tromsø, Norway; 3 Department of Pharmacy, University of Oslo, Oslo, Norway; 4 Hospital Pharmacy of North Norway Trust, Tromsø, Norway; 5 Municipality of Tromsø, Tromsø, Norway; 6 Nøste Private Healthcare Centre, Lier, Norway; 7 Department of Geriatric Medicine, University Hospital of North Norway, Tromsø, Norway

**Keywords:** Clinical pharmacist intervention, Hospital, Medication safety, Older adults, Randomized controlled trial, Integrated medicines management

## Abstract

**Background:**

Suboptimal medication use contributes to a substantial proportion of hospitalizations and emergency department visits in older adults. We designed a clinical pharmacist intervention to optimize medication therapy in older hospitalized patients. Based on the integrated medicine management (IMM) model, the 5-step IMMENSE intervention comprise medication reconciliation, medication review, reconciled medication list upon discharge, patient counselling, and post discharge communication with primary care. The objective of this study was to evaluate the effects of the intervention on healthcare use and mortality.

**Methods:**

A non-blinded parallel group randomized controlled trial was conducted in two internal medicine wards at the University Hospital of North Norway. Acutely admitted patients ≥ 70 years were randomized 1:1 to intervention or standard care (control). The primary outcome was the rate of emergency medical visits (readmissions and emergency department visits) 12 months after discharge.

**Results:**

Of the 1510 patients assessed for eligibility, 662 patients were asked to participate, and 516 were enrolled. After withdrawal of consent and deaths in hospital, the modified intention-to-treat population comprised 480 patients with a mean age of 83.1 years (SD: 6.3); 244 intervention patients and 236 control patients. The number of emergency medical visits in the intervention and control group was 497 and 499, respectively, and no statistically significant difference was observed in rate of the primary outcome between the groups [adjusted incidence rate ratio of 1.02 (95% CI: 0.82–1.27)]. No statistically significant differences between groups were observed for any of the secondary outcomes, neither in subgroups, nor for the per-protocol population.

**Conclusions:**

We did not observe any statistical significant effects of the IMMENSE intervention on the rate of emergency medical visits or any other secondary outcomes after 12 months in hospitalized older adults included in this study.

**Trial registration:**

The trial was registered in clinicaltrials.gov on 28/06/2016, before enrolment started (NCT02816086).

**Supplementary Information:**

The online version contains supplementary material available at 10.1186/s12913-022-08648-1.

## Background

Medications have a pivotal role in improving the quality of life and preventing morbidity and mortality, but are also an important cause of patient harm, especially in older adults [1, 2]. A medication-related problem (MRP) is defined as 'an event or circumstance involving drug therapy that actually or potentially interferes with desired health outcomes [3, 4]. Among older adults, 10–20% of hospitalizations are caused by MRPs [5–9] and possibly more in patients with multimorbidity or dementia [10, 11]. A large proportion of these medication-related hospitalizations may be preventable [5, 6, 8].

Providing clinical pharmacist services in hospitals, such as medication reconciliation, medication review, and patient counselling can reduce the number of medication discrepancies, identify, and solve MRPs, improve medication appropriateness, and improve adherence [12–16]. However, studies investigating the effects of clinical pharmacist services on patient outcomes such as readmissions and emergency department (ED) visits have shown conflicting results [16, 17]. Systematic reviews suggest that multifaceted interdisciplinary interventions with pharmacists as integrated team members may be necessary for interventions to impact patient outcomes [16, 18, 19].

The integrated medicines management (IMM) model is an interdisciplinary intervention for which reduced rate of readmissions, increased time to readmission, and increased overall survival have been shown [13, 20–22]. The IMM model systematically integrates medication reconciliation, medication review, patient counselling and dissemination of correct medication information at transition points, holding clinical pharmacists as key team members [13, 20]. However, there are conflicting results on patient outcomes. A recently published randomized controlled trial (RCT) from Norway found no significant effects on readmissions in hospitalized multimorbid patients [22]. As older patients are particularly vulnerable to new hospitalizations in the time after discharge, bridging the transitions across secondary and primary care may be an important element in interventions aiming to reduce hospital visits [23].

Based on the IMM model, we designed an interdisciplinary intervention aiming to improve communication with health care workers in primary care. The primary aim of the randomized controlled trial IMMENSE (IMprove MEdicatioN Safety in the Elderly) was to investigate the effects of the intervention on the rate of emergency medical visits (readmissions and ED-visits) 12 months after discharge in older inpatients [24]. Secondary aims were to investigate its impact on i) the length of index hospital stay ii) time to first acute readmission, iii) the proportion of patients readmitted acutely within 30 days and iv) mortality rate during the same period.

## Methods

### Study design

This is a parallel group non-blinded RCT with an intervention group and a control group (1:1 ratio). Study enrollment started in September 2016 and ended in December 2019. All patients were followed up for 12 months after hospital discharge.

The trial was conducted in compliance with the published study protocol [24], the principles of Good Clinical Practice and the Declaration of Helsinki and is reported according to The Consolidated Standards of Reporting Trials (CONSORT) reporting guideline and template for intervention description and replication (TIDieR) checklist [24–27].

### Settings and participants

The study was carried out at a geriatric internal medicine ward and a general internal medicine ward at the University Hospital of North Norway (UNN). The geriatric ward cares for older patients with complex acute medical needs, and physicians are specialized in geriatric medicine. The general medicine ward treats patients admitted for stroke, pulmonary-, kidney- and endocrine diseases as well as patients with geriatric concerns. Pharmacists were not involved in standard patient care at the study wards.

Inclusion criteria were acutely admitted patients aged ≥ 70 years and willing to provide written informed consent (patient or next of kin). Patients were excluded if they had been admitted to the study ward more than 72 h before evaluation of eligibility, moved to and discharged from other wards during the index stay, unable to understand Norwegian (patient or next of kin), considered terminally ill or with a anticipated short life expectancy, were planned discharged on the inclusion day, occupying a bed in a study ward but under the care of physicians from a non-study ward, or if intervention from a study pharmacist was considered necessary for ethical reasons (before randomization or in the control group). Readmitted study patients were not re-included due to limited pharmacist resources, but received standard care. Patients referred to a patient-centred care team project upon discharge, including pharmaceutical care, were not excluded.

Patients were screened for eligibility and recruited by study pharmacists. Enrolment and clinical work were performed from 8.00 am—3.30 pm on weekdays. In the geriatric ward, the study pharmacists were present every weekday, but only every other weekday in the general medicine ward. Patients were approached for inclusion in a predetermined order to avoid selection bias.

### Randomization and blinding

After collecting baseline data, patients were randomized by study pharmacists using a web-based service supplied by the department of applied clinical research at the Norwegian University of Science and Technology. The randomization block sizes were permuted, of unknown and variable size and stratified by the study site. As pharmacists were only involved with patients in the intervention group, blinding of group allocation for patients, pharmacists, and the interdisciplinary team was impossible. However, the primary analysis was performed by an investigator not involved in the data collection and blinded for group allocation (KS).

### The intervention and standard care

The intervention was based on the IMM model, including a pharmacist in the interdisciplinary ward team working closely with the patients, physicians, and other team members [24]. Briefly, the five-step IMMENSE intervention comprised medication reconciliation, medication review, medication counselling, transmission of medication information upon discharge and finally, oral communication with primary care after discharge, see Table 1. Control group patients received standard care, which was care from the same ward team, except the services provided by the pharmacist. Six pharmacists were involved in delivering the intervention throughout the study period, all holding master's degrees in pharmacy and trained in the IMM study procedures.

**Table 1:** Description of the IMMENSE intervention steps with corresponding activities in standard care

	Description Intervention	Description standard care
Step 1: Medication reconciliation	If possible, pharmacists interviewed patients about their ongoing medications, applying a standardized IMM medication reconciliation interview, including questions about medication use, practical handling, knowledge and medication adherence. Information about the patients' medicines use was also collected from other relevant sources, and a best possible medication list was compiled. This list was then compared to the medication list in use at the ward at study inclusion and medication discrepancies were discussed with the physicians and corrected	As part of the national patient safety program, medication reconciliation should be performed by a physician at admission and the sources used in the reconciliation process documented in the patient journal
Step 2: Medication review	A standardized IMM procedure was applied. The structured and comprehensive medication review identifies MRPs in ten prespecified risk categories. Identified MRPs were discussed in the interdisciplinary team and with patients if possible, the physician being in charge of medication changes. The medication review was performed at study inclusion and updated regularly during the hospital stay when the study pharmacists were present at the ward	Medication reviews performed by physicians are a part of standard care, especially in the geriatric ward, however it is not standardized or structured
Step 3: Medication list in discharge summaries	The study pharmacists drafted medication lists in the electronic medical journals that were reconciled, structured and correct. The medication lists included information and explanations about medication changes made during the hospital stay and unsolved MRPs with recommendations, as well as needs for monitoring of medication therapy. This information was used by the responsible ward physician to compile the final discharge summary to be submitted to the primary care physicians	Local procedures for communication of medication information at hospital discharge require that a discharge summary, including updated medication lists in addition to assessments, amendments and recommendations made during the hospital stays, are submitted electronically to the GP upon discharge. This is the responsibility of the physician
Step 4: Patient counselling	A patient counselling session with the study pharmacist was arranged before discharge for patients who would handle their own medication after discharge. The patients received an updated medication list, which was discussed and explained. In the counselling, the pharmacists focused on changes made during the hospital stay and the reasons for these changes. Patients were encouraged to ask questions about their medications	Physicians normally talk to all patients upon discharge; the focus on medications depends on the physicians´priorities and the patients' needs
Step 5: Communication with primary care	Within a week after discharge, the pharmacists called the patients' GP to discuss medication therapy changes made in hospital, as well as recommendations and monitoring needs stated in the discharge summary (if relevant). The aim was to ensure that the changes and recommendations were implemented. Upon discharge, the pharmacists or ward nurses called home care services or nursing homes if these were responsible for administering the patients' medications. Medication changes were discussed, and multi dosage dispensed medications changed in agreement with home care services	Oral communication with GPs upon discharge is not part of standard care. For patients living in nursing homes or cared for by the home care services, ward nurses often call to investigate the need for prescriptions or medications to be sent home with the patients

*GP* general practitioner, *IMM* integrated medicines management, *MRP* Medication-related problem

### Primary and secondary outcomes

The primary outcome was the rate of emergency medical visits 12 months after discharge from the index hospital stay, an endpoint relevant both for patients and health care systems, previously shown to be affected by similar interventions in similar health care systems, e.g. in the study by Gillespie et al. [28]. Emergency medical visits is a composite outcome of acute readmissions and ED visits. We defined acute readmissions as any subsequent admission following the index stay, excluding elective readmissions. ED visits included emergency visits to the hospital and visits to municipality-run emergency medical clinics if the patients were not subsequently admitted to the hospital. A prespecified secondary analysis of the time to reach the primary outcome and the proportion of patients reaching the primary outcome was performed.

Secondary outcomes included i) the length of index hospital stay ii) time to first acute readmission, iii) the proportion of patients readmitted acutely within 30 days and iv) mortality rate during 12 months of follow-up. Other prespecified outcomes relating to inappropriate prescribing, medication-related readmissions and health-related quality of life specified in the study protocol will be addressed in future articles.

### Data collection and outcome assessment

Baseline data collected: age, gender, marital status, level of education, type and amount of help from home care services, delivery of multi dosage dispensed medications, medical diagnosis/medical history, and medication use at the time of hospital admission. Data was registered in a Microsoft® Access database which has been the basis for previously reported data on intervention fidelity and process data on identified MRPs [29].

Data on outcomes was collected from national health registries; readmissions and hospital ED visits from The Norwegian Patient Registry, emergency medical visits to EDs run by local municipalities from The Norwegian Health Economics Administration Registry, and deaths from the National Cause of Death Registry [30]. Linking data was possible through the unique personal identification number assigned all Norwegian citizens. An ED visit within the six-hour window before a hospital stay was counted as a hospital stay only. We collected registry data from 12 months before and 12 months after the index stay to enable adjustments for pre-study risk factors.

### Sample size calculation

Sample size calculation for the primary outcome was based on the study by Gillespie et al. applying the same composite endpoint [28]. This trial investigated the effectiveness of a multifaceted intervention including post-discharge interventions performed by ward-based pharmacists in reducing morbidity and hospital visits among patients 80 years and older. They randomized 400 patients in a 1:1 relationship and found a 16% reduction in all-cause visits to the hospital in the intervention group. We estimated a rate of acute hospital admissions and ED visits of 1.7 per year in our patient population. Consequently, we needed to enrol 456 patients (228 in each group) to detect a 16% reduction in hospital visits with a 5% significance level and 80% power. Taking dropouts into account, we aimed to include 250 patients in each group. We extended the enrollment period three weeks after reaching 500 patients to compensate for exclusions.

### Statistical analysis

Data was analyzed by an intention-to-treat (ITT) principle but modified as registry data on endpoints were unavailable for patients who withdrew the informed consent. We also excluded patients dying during the index hospital stay from the analysis. The statistical analysis plan (SAP) can be found in Supplement 1. A prespecified per-protocol (PP) analysis, including patients not excluded after randomization, was also performed.

The primary analysis was a multilevel Poisson regression to handle clustering on the study ward level and repeated measurements on the patient level. We applied time out of hospital alive (days at risk of an event) in the 365 days after discharge as an offset and adjusted for the number of emergency medical visits in the 365 days prior to the index hospitalization.

Time to first readmission and time to first emergency medical visit was analyzed by the Kaplan–Meier method and the log-rank test. A Cox proportional Hazards Model (adjusted and unadjusted) was applied to estimate hazard ratios (HRs), which are presented with 95% confidence intervals (CIs). The differences in lengths of stays between groups were assessed with an independent sample Mann–Whitney test. The differences in proportions of patients alive at 12 months and patients readmitted within 30 days were compared with logistic regression (adjusted and unadjusted). A two-sided alpha level of 5% with no adjustments for multiplicity was used as a statistical significance level.

The effect of the intervention on the primary endpoint was explored in the following prespecified subgroups i) number of medications upon admission or discharge; 0–5, 6–10, > 10, ii) age groups; 70–80, 80–90 and > 90, iii) patient responsible for their own medication after discharge; yes, no, partly, iv) Charlson Comorbidity Index score; 0–2, > 2, v) the number of hospital visits in the 12 months prior to inclusion; 0–1, > 1, vi) length of hospital stay; 0–6 days, > 6 days, vii) living status before hospitalization; referred from home, home-care or nursing home, and viii) ability to self-provide informed consent or not.

The multilevel Poisson regression was performed in STATA® 16.1, data management and the remaining analyses in IBM® SPSS Statistics Version 28.

## Results

**Fig. 1:**
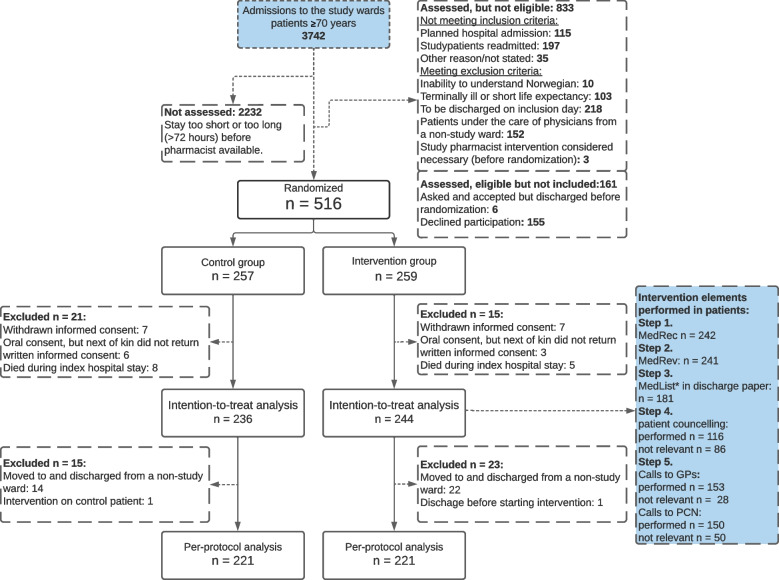
Flow diagram of patients included in the IMMENSE study. Abbreviations: MedRec; medication reconciliation, MedRev; medication review, MedList: medication list, GP; general practitioner, PCN; Primary care nurses, * medication list according to study procedures in the discharge papers

During the enrollment period, 3742 patients ≥ 70 years were admitted to the two study wards, 1510 were assessed for eligibility and 662 were asked to participate. Out of the 516 who consented, 257 were randomized to the control group and 259 to the intervention group, see Fig. 1. The rate-limiting step of the inclusion process was the pharmacists' capacity to screen and include patients while working with study patients. Consequently, many patients were discharged or admitted for > 72 h (exclusion criterion) before they could be screened or invited to participate. Of the 516 patients included, 23 patients withdrew consent and 13 died during hospitalization, leaving 480 patients in the ITT population, see Table 2 for baseline characteristics. The PP population comprised 442 patients, as 38 patients were transferred and discharged from non-study wards and consequently excluded from the ITT population, see Supplement 2, Table 1 for baseline characteristics.

**Table 2:** Baseline characteristics of the ITT population (*N* = 480)

Characteristics	Intervention group *n* = 244		Control group *n* = 236		
Age, mean years (SD)	83.3	(6.4)	83.0	(6.3)	
Sex, female, n (%)	152	(62.3)	127	(53.8)	
Study Site, n (%)					
Geriatric ward (study site 1)	198	(81.1)	191	(80.9)	
General medicine ward (study site 2)^a^	46	(18.9)	45	(19.1)	
Ability to self-provide consent, n (%)	174	(71.3)	160	(67.8)	
Marital status, n (%)					
Widow/widower	107	(43.9)	104	(44.1)	
Married/live in partnership	101	(41.4)	88	(37.2)	
Single/ Divorced/separated	34	(13.9)	41	(17.4)	
Missing	2	(0.8)	3	(1.3)	
Educational level, ISCED level^b^ n (%)					
Elementary school, level 1	107	(43.9)	109	(46.2)	
Lower/upper Secondary education, level 2–3	93	(38.1)	81	(34.3)	
Higher education (< 4 years), level 5–6	22	(9.0)	18	(7.6)	
Higher education (> 4 years), level 7–9	11	(4.5)	12	(5.1)	
Missing	11	(4.5)	16	(6.8)	
Living status upon admission, n (%)					
Home, no help from home care services	88	(36.1)	69	(29.2)	
Home, with help from home care services	116	(47.5)	139	(58.9)	
Nursing home, short term	22	(9.0)	13	(5.5)	
Nursing home, permanent	18	(7.4)	15	(6.4)	
Discharge to home, n (%)	151	(61.9)	132	(55.9)	
Handling medications themselves, n (%)					
Yes	94	(38.5)	80	(33.9)	
No	104	(42.6)	101	(42.8)	
Partly	46	(18.9)	54	(22.9)	
Missing	0		1	(0.4)	
Co-morbidity^c^ (median score, IQR)					
Charlson comorbidity index	2	(1–3.75)	2	(1–4)	
Number of medications (ATC-codes) in use at hospital admission, Median (IQR)					
Total	8	(5–12)	9	(6–13)	
Regular use	6	(4–9)	7	(4–10)	
Use as needed	2	(0–3)	2	(0–3)	
Medical history in admission notes, n (%)					
Hypertension	125	(51.5)	113	(47.9)	
Atrial fibrillation	67	(27.5)	65	(27.5)	
Asthma or COPD	55	(22.5)	53	(22.5)	
Diabetes Mellitus	50	(20.5)	52	(22.0)	
Heart failure	40	(16.4)	36	(15.3)	
Dementia	34	(13.9)	32	(13.6)	
Emergency medical visits, one year before index hospital stay					
Emergency medical visits, n (% with ≥ 1)	462	(68.4)	548	(72.5)	
Emergency medical visits, median (IQR)	1	(0–3)	1	(0–3)	

Abbrevations: *ATC* anatomical therapeutic chemical classification system, *IRQ* interquartile range, *ISCED* international standard classification of education, *SD* standard deviation

^a)^ no specialized geriatric ward existed at this hospital, but four beds in the study ward was dedicated to patients with geriatric concerns

^b)^ educational level categorized by the international standard classification of education [31]

^c)^ co-morbidity based on diagnosis found in admission and discharge papers from index admission, calculated in accordance with Charlson et al. [32]

The groups were well balanced at baseline, but control group patients received more regular medications, more help in their home, and had more emergency medical visits in the year before index stay. Medication reconciliation and medication review were provided to all but three patients. Step 3, 4 and 5 were received by 74–83% of patients where the procedures were relevant (see Fig. 1). See Johansen et al. for further details on intervention fidelity and process outcomes (MRPs and medication discrepancies) of the PP population [29].

After 12 months, the number of emergency visits was 497 in the intervention group and 499 in the control group, with a non-significant adjusted IRR of 1.02; 95% CI: 0.82–1.27 (Table 3). No significant differences were identified in the subgroup analyses (Supplement 2, Table 2). We explored selection and time-related biases in two post hoc analyses. Contamination bias due to that patients referred to a patient-centred health care team delivering clinical pharmacist services was explored by excluding the 32 control and 32 intervention patients referred to this team from the main analyses. We observed no change in risk estimate of the primary outcome (adjusted IRR 1.08, 95% CI: 0.85–1.38). Time-dependent bias was explored by running the main analysis only including the first 240 or the last 240 included patients. In this analysis, risk estimates changed slightly, but not significantly, from IRR 0.92 (95% CI 0.68–1.25) in the first 240 patients to IRR 1.13 (95% CI 0.83–1.53) in the last 240 patients.

**Table 3:** Primary and secondary outcomes in the ITT population (*N* = 480)

Primary outcome after 12 months	Intervention		Control			
	(*n*=244)		(*n*=236)		Crude	Adjusted^a^
	n, median (IQR)		n, median (IQR)		Incidence rate ratio (95 % CI)	
Emergency medical visits	497	1 (0-3)	499	1 (0-3)	0.95 (0.75-1.20)	1.02 (0.82-1.27)
ED-visits	277	1 (0-2)	276	1 (0-2)	0.95 (0.72-1.26)	1.02 (0.78-1.33)
Readmissions	220	1 (0-1)	223	0 (0-1.75)	0.96 (0.73-1.25)	1.01 (0.78-1.30)
**Secondary outcomes**						
Days to first event	median (%)		median (%)		Hazard rate (95 % CI)	
Emergency medical visit	137	(71.3)	110	(70.3)	0.93 (0.75-1.15)	0.96 (0.78-1.19)
Readmission	310	(50.8)	356	(47.5)	1.05 (0.81-1.35)	1.10 (0.85-1.42)
	n (%)		n (%)		Odds ratio (95 % CI)	
Readmissions within 30 days	26	(10.7)	33	(14.0)	0.73 (0.42-1.27)	0.82 (0.46-1.44)
All-cause mortality within 12 months	48	(19.7)	46	(19.5)	1.01 (0.64-1.59)	1.06 (0.67-1.69)

*IQR* Interquartile Range

^a)^ Adjusted for the number of emergency medical visits during 365 days prior to the index hospital stay

**Fig. 2:**
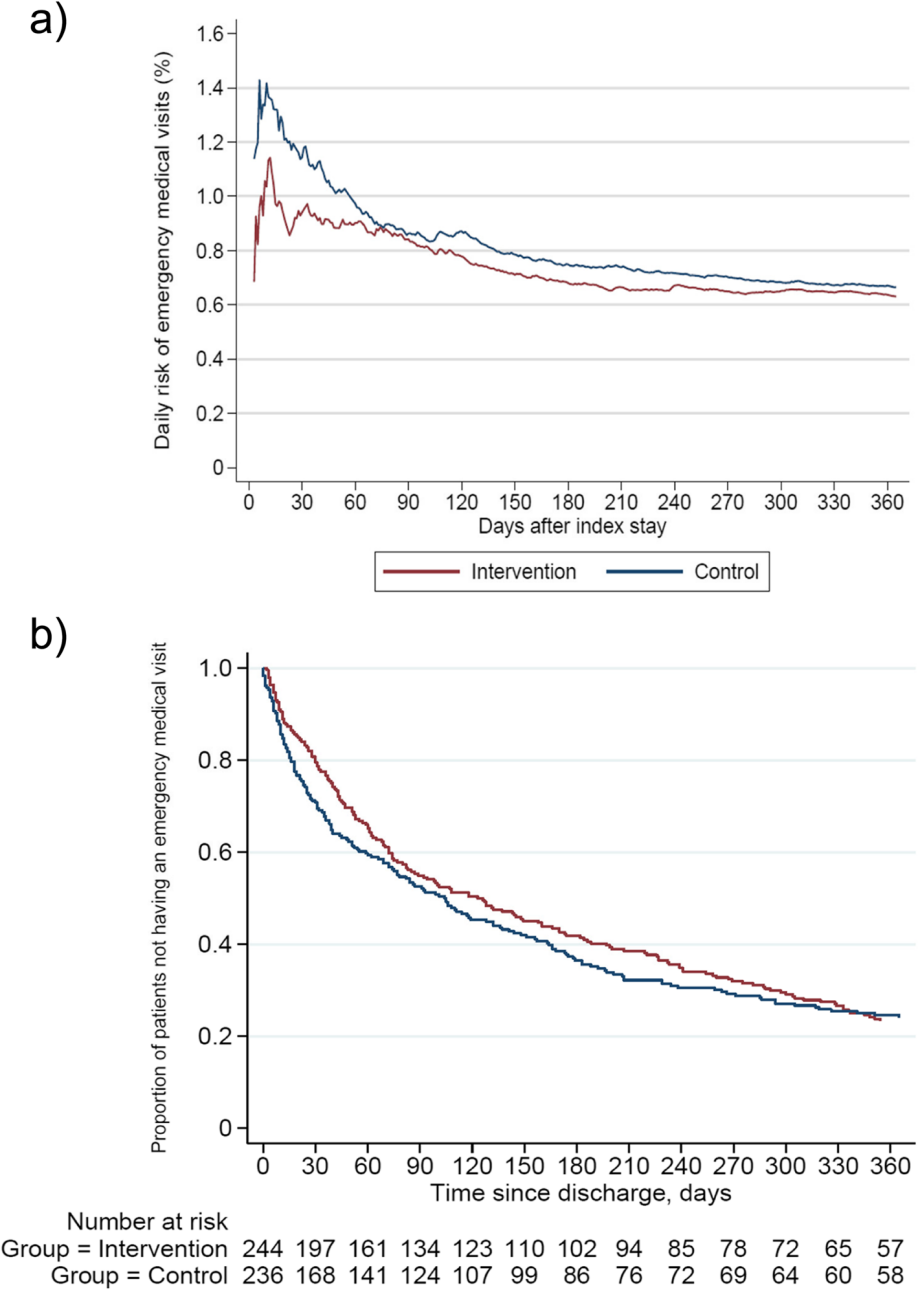
Emergency medical visits in the ITT-population (*N* = 480) illustrated by **a**). The daily risk of new emergency medical visits and **b**). Kaplan–meier plot of time to first emergency medical visit

Daily risk of emergency medical visits appeared to be higher in the control group the first two months after discharge (Fig. 2a). Still, these differences after 30 days were not significant when controlling for the rate of emergency visits in the year before the index hospital stay, with an adjusted IRR of 0.77 (95% CI 0.48 – 1.44).

The secondary outcomes are presented in Table 3; no significant differences between the groups were identified. Although not statistically significant, the Kaplan Meier plot of time to first emergency medical visit (Fig. 2b) slightly favours the intervention group over the control group, 137 days vs 110. On the other hand, median time to first hospital readmission was lower in the intervention group with 310 days compared to the control group with 356 days, adjusted HR of 1.1; 95% CI 0.85–1.42. The median length of the index hospital stay was similar in the intervention vs control group [median 6 (IQR:4–9) vs 6 (IQR:3–11) *p* = 0.536]. No significant differences were identified for any of the outcomes in the PP population, although the risk estimates moved slightly in favour of the intervention group (Supplement 2, Table 3).

## Discussion

In this trial, we observed no significant effect of the 5-step IMMENSE intervention on the rate of emergency medical visits 12 months after discharge in hospitalized older adults compared to standard care. Nor did we observe any significant effects on secondary outcomes related to healthcare use and mortality. The lack of observed effects is likely multifactorial, influenced by factors such as intervention complexity and content, intervention delivery, choice of endpoints, patient population, study context, healthcare team collaboration, development of standard care over the period and acceptability by patients and collaborators.

Our results are in line with two other RCTs performed simultaneously in Scandinavia [22, 33]. Both studies failed to show a significant reduction in readmissions or ED visits after 12 months, despite applying multifaceted interdisciplinary interventions with pharmacists as integrated team members as recommended [16, 18, 19]. One possible explanation for the lack of effect of the IMMENSE intervention, may be that the intervention elements were not patient-focused enough. A Danish study by Ravn-Nielsen et al. found that a pharmacist-led intervention in hospitals, including motivational interviews (MI) and postdischarge follow-up with patients and primary care, significantly reduced the risk of hospital readmission after six months [34]. MI is well-known as a method of councelling patients, and has proved useful in the treatement of lifestyle problems and diseases [35]. It has shown positive effects for several health-related outcomes [35], and in pharmacy practice, MI has e.g., demonstrated to improve medication adherence [36]. In the IMMENSE study, MI was not part of the intervention, nor was it in the cluster RCT by Kempen et al. where hospital-based comprehensive medication reviews, including postdischarge follow-up of older patients failed to reduce hospital vistis in the year after discharge [33]. Patient-focused intervention elements may prove important to increase the impact of similar pharmacist-led interventions. However, considering the high mean age in the IMMENSE population, this does not seem to be the entire explanation.

A second possible explanation for the lack of effect may be a failure to implement the intervention properly, which we do not fully believe is the case. The intervention fidelity of the IMMENSE intervention was generally good, as the first two steps of the intervention was delivered to all but three participants, and the remaining steps to more than 70% of the participants where these were relevant [29]. The steps relating to discharge and communication with primary care were the most challenging to implement, requiring time from collaboration health care personnel. However, our implementation rates are in line with similar studies [22, 33, 37]. In addition, several MRPs (median 4, IQR 2–6) and medication discrepancies (median 1, IQR 0–3) were identified among intervention patients [29]. In total, 67% of MRPs were solved in the interdisciplinary team in the hospital as recommended by the pharmacist, and 23% communicated to primary care [29], suggesting a high agreement within the interdisciplinary hospital team and a realistic potential for optimized medication regimes. Despite these positive figures, health care use was not significantly affected.

A third possible explanation for the lack of effect may be the choice of endpoint. In retrospect, it may be optimistic to expect a one-time delivered intervention in hospital to affect future acute hospital visits in such an old, multimorbid, and help-demanding population over such a long period of time. From our data on the risk of new events over time (Fig. 2a), we did identify a small but non-significant difference between the two study groups in the first few months after discharge, which was not present after 12 months. Also, as we know that medication changes occur frequently in older adults after hospital discharge [38], it would be expected for any potential effect of the intervention to taper off when no new intervention is provided [39, 40]. Providing repeated interventions upon readmissions should be considered. We had aimed at promoting sustainable effects of the intervention by including the fifth step of the IMMENSE intervention, enabling the GPs to improve follow-up on medication optimization. How GPs acted upon the recommendations is however unknown [29]. In future studies, interventions in hospitals should consider even closer collaboration with primary care, including follow-up at home or in primary care centers. Also a shift towards more patient-focused outcomes should be considered [41]. This is confirmed by stakeholders in the study by Beuscart et al. from 2018, developing a core outcome set for clinical trials of medication reviews in multimorbid older patients with polypharmacy [42]. The only healthcare-related outcome considered as a core outcome by stakeholders, was medication-related hospital admissions [42]. In the current study, the effects of the intervention on the more patient-focused outcomes as health-related quality of life, potentially inappropriate prescribing, and medication-related readmission remains to be established [24].

A fourth explanation to the lack of effect of the IMMENSE intervention may be the development in standard care over the last decade. This was also an argument made by Kempen et al. related to the Swedish cluster RCT, where they failed to reproduce the positive 16% reduction in hospital visits demonstrated by Gillespie et al. 12 years earlier [28, 33]. In Norway several initiatives have been taken to improve medication use in older adults, like national campaigns on medication reconciliation [43], regulations for GPs to perform medication reviews [44], developments in electronic communications between care levels, and national summary care records [45]. These developments will likely decrease the differences between the intervention group and the standard care group, making results of previous studies hard to replicate.

Compared to findings in the recent Norwegian study published study by Lea et al., where a significant reduction in 20 months all-cause mortality was observed [22], other explanations to the lack of effect of the IMMENSE intervention may also be relevant. Despite the similarity between the two interventions, Lea et al. had a longer follow-up time for outcomes, used pharmacists with post-graduate degrees in clinical pharmacy and identified more MRPs [29]. The mortality rates in the study population was also higher compared to the IMMENSE population, suggesting differences between the two study populations. Moreover, the study settings differed as the study by Lea et al. was performed in an internal medicine ward, whereas 77% of patients in the IMMENSE study was recruited from a specialized geriatric ward. In geriatric wards, health care personnel tend to take a more active approach towards medication optimization than other internal medicine wards [46], possibly reducing the effects of the tested intervention. However, in our study, the subgroup analysis (Supplement 2, Table 2) did not identify statistically significant effects in either study site (geriatric vs. general medical ward).

### Strengths and limitations

This study has several strengths such as the randomized controlled design to create comparable study groups and control for bias, and the blinding of the investigator performing the primary analyses. Furthermore, the Norwegian health registries enable a complete and quality assured collection of outcomes. The collection of data for the 365 days prior to the index hospital stay, enabled us to adjust for pre-study patterns. This was important as the control group seemed to be somewhat sicker at baseline and controlling for the rate of the primary endpoint in the year prior gave strength to the analysis. Finally, including patients with dementia and cognitive impairment, increase the generalizability of findings.

There are also limitations that need to be addressed. First, intervention and control patients were included from the same wards and cared for by the same health professionals, which may have introduced a contamination bias, reducing between-group differences. This could have been prevented by including more wards and performing cluster-randomization, but was not practically possible due to limited funding. Second, the inclusion rate was slow as the pharmacists were only able to include a limited number of patients each day due to the workload associated with study-related tasks and delivering the intervention [47]. Consequently, a small proportion of admitted patients were screened for eligibility or asked for participation, possibly introducing a selection bias. To prevent selection bias, the study pharmacists always approached patients in a predetermined order (last-admitted-asked first). Third, due to a slow inclusion rate, the enrollment period lasted for three years, which enabled changes in standard care at the wards related to medication management, e.g., new methods for medication reconciliation. How changes in standard care may have influenced the study results is unknown, however it does not seem very likely from our post-hoc analyses. Fourth, our study was powered to investigate a possible difference in number of events after one year. In retrospect, we recognize that the intervention was probably underpowerd to identify a more likely effect on short-term event rates. Finally, due to the complexity of the intervention, not all intervention steps were delivered to all patients, and whether recommendations were followed-up upon in primary care is unknown [29]. A process evaluation alongside the trial could have enabled the identification of barriers and enablers to the effective delivery of the intervention, which would have provided valuable information on how to develop better interventions in the future [48].

## Conclusion

We did not observe a statistically significant effect of the IMMENSE intervention on the rate of emergency medical visits after 12 months or any of the other secondary outcomes in hospitalized older adults included in this study. The study adds to recent evidence suggesting that outcomes related to reductions in healthcare use are not sensitive to the effects of hospital-based clinical pharmacist services. However, these interventions are complex, and their ability to affect outcomes depends on numerous factors. Future studies should incorporate process evaluations alongside the trial to explain the factors that may influence study outcomes. This may enable us to design better and more effective interventions in the future.

## Supplementary Information

**Additional file 1.** 

**Additional file 2.** 

## Data Availability

The data that support the findings of this study are available upon reasonable request from the corresponding author JSJ. The data are not publicly available due to them containing information that could compromise research participants´ privacy/consent.

## Abbreviations

CI: Confidence Interval
CONSORT: The Consolidated Standards of Reporting Trials
ED: Emergency Department
GP: General Practitioner
HR: Hazards Ratio
IMMENSE: IMprove MEdicatioN Safety in the Elderly
IMM: Intergrated medicines management
IQR: Interquartile Range
IRR: Incidence Rate Ratio
ITT: Intention-to-treat
MI: Motivational interviewing
MRP: Medicine-related Problem
OR: Odds Ratio
PP: Per-protocol
RCT: Randomized Controlled Trial
SD: Standard Deviation
TIDieR: Template for intervention description and replication
UiT: University of Tromsø
UNN: University Hospital of North Norway
