# Supplementary material for: Interdisciplinary collaboration across secondary and primary care to improve medication safety in the elderly (The IMMENSE study) – a randomized controlled trial
Source: BMC Health Serv Res. 2022 Oct 26;22:1290. doi: 10.1186/s12913-022-08648-1 (PMC9597977; doi:10.1186/s12913-022-08648-1)
Supplement: Supplementary file 1 — Additional file 1. [file 12913_2022_8648_MOESM1_ESM.pdf]

Statistical Analysis Plan (SAP)

TRIAL FULL TITLE	A new interdisciplinary collaboration structure in secondary and primary care to improve medication safety in the elderly (IMMENSE study) – a randomized controlled trail
Protocol	https://bmjopen.bmj.com/content/8/1/e020106
PVO NUMBER	41366<br>The Norwegian Centre for Research Data and the Norwegian Data Protection Authority
SAP VERSION	1.0
CLINICAL TRIAL NUMBER	NCT02816086
SAP VERSION DATE	2021-03-18
TRIAL STATISTICIAN	Frode Skjold
TRIAL CHIEF INVESTIGATOR	Beate H. Garcia
SAP AUTHORS	Beate H. Garcia, Jeanette Schultz Johansen, Kjell H. Halvorsen and Frode Skjold

This SAP will concern the statistical analysis on the primary and secondary endpoints of the main article of the intervention study. Some secondary endpoints mentioned in the protocol article is excluded and will be presented in future SAPs.

SAP Signatures

I give my approval for the attached SAP entitled “A new interdisciplinary collaboration structure in secondary and primary care to improve medication safety in the elderly (IMMENSE study) – a randomized controlled trial” dated xx.xx.2020

Chief Investigator

Name: Beate H. Garcia

Signature: Beate Garcia
Date: 18.03.2021

Statistician

Name: Frode Skjold

Signature: Frode Skjold
Date: 18.03.2021

Abbreviations

CCI	Charlson Comorbidity Index
ED	Emergency Department
EQ5D-VAS	EuroQoL 5L - health-related quality of life
GP	General practitioner
HRQoL	Health related quality of life
MMS	Mini-mental Status
SAP	Statistical Analysis Plan
TILT	No; Tidlig Identifisering av Livstruende Tilstander<br>Eng; Early Identification of Life-threatening Conditions.

Definitions

An "acute readmission" is defined as when a patient unplanned has been formally admitted to a hospital ward, independent whether the patient was visiting the ED before hospitalization.

An "ED visit", is defined as when a patient unplanned have been visiting the ED (including both the ED run by the municipality and the ED run by the hospital) but not formally admitted to the hospital. If the patient is admitted to hospital following an ED visit, it will be defined as a readmission.

Emergency department (ED) In Norway, the medical emergency service is divided in two; one run by the hospital (only localized in towns where there is a hospital) and one run by the municipalities (also localized in towns where there is a hospital and consequently a hospital-run ED). Patients are not supposed to arrive in the hospital-run ED without a referral from their GP, the municipality-run ED or arriving with the ambulance.

The EDs run by the municipality are employed by general practitioners (GPs) and open when the GPs´ offices are closed, normally 4 pm – 8 am. The EDs run by the hospital are open 24/7.

TABLE OF CONTENTS

1. BRIEF INTRODUCTION 4

2. STUDY OBJECTIVES AND ENDPOINTS 4

2.1 OBJECTIVES 4
2.2 PRIMARY ENDPOINT 4
2.3 SECONDARY ENDPOINTS 5

3. METHODS 5

3.1 GENERAL STUDY DESIGN AND PLAN 5
3.2 INCLUSION-EXCLUSION CRITERIA AND GENERAL STUDY POPULATION 5
3.3 RANDOMISATION AND BLINDING 6
3.4 SAMPLE SIZE 6

4 GENERAL CONSIDERATIONS 6

4.1 TIMING OF ANALYSES 6
4.2 ANALYSIS POPULATIONS 6
4.3 VARIABLES, DATA SOURCES AND SUBGROUPS 6
4.4 MISSING DATA 7
4.5 MULTI-CENTRE STUDIES 7
4.6 MULTIPLE TESTING 7
5.1 PATIENT FLOW 8
5.2 PROTOCOL DEVIATIONS 9
5.3 DEMOGRAPHIC AND BASELINE VARIABLES 9
5.4 CONCURRENT ILLNESSES AND MEDICAL CONDITIONS 10
5.5 PRIOR AND CONCURRENT MEDICATIONS 10
5.6 TREATMENT COMPLIANCE (INTERVENTION FIDELITY) 10

6 EFFICACY ANALYSES 10

6.1 GENERAL ON STATISTICS 10
6.2 INTENTION TO TREAT AND PER PROTOCOL ANALYSIS 10
6.3 PRIMARY ENDPOINT ANALYSIS 11
6.4 SECONDARY ENDPOINT ANALYSIS 13
6.5 BLINDING 13

7 SUMMARY OF CHANGES TO THE PROTOCOL 14

8 REFERENCES 14

APPENDIX 1: VARIABLES COLLECTED FOR USE IN PRIMARY AND SECONDARY ANALYSES 15

1. Brief introduction

This non-blinded randomized controlled trial investigates whether an interdisciplinary intervention in geriatric patients (>70y) admitted to hospital will impact patient outcomes. The intervention is a new inter-professional collaboration structure between hospital physician, pharmacists and GPs focusing on medications applying the Integrated Medicines Management (IMM) methodology. The novelty is the inclusion of the clinical pharmacist in the team, who performs medication reconciliation, medication review, ensures correct communication about medications to patients and primary care and follows up with primary care after discharge. The study includes patients from two hospital sites; a geriatric ward at the University Hospital of North Norway (UNN) Tromsø and a general internal medicines ward at UNN, Harstad (1).

Following in the document, amendments from the published protocol will be pinpointed and ambiguities in the protocol descriptions will be clarified, see grey boxes.

2. Study Objectives and Endpoints

2.1 Objectives

The primary objective is to investigate the effects of the intervention on rate of emergency medical visits (acute readmissions and visits to emergency departments (EDs)) 12 months after hospital discharge.

Secondary objectives include to investigate the effects on:

- Acute readmissions
- Length of index hospital stay
- Time to first readmission
- 30-day readmissions
- Mortality rate

Specification:
- Regarding the primary objective, "Rate of emergency medical visits" is referring to the number of emergency medical visits per patient included in the trial.
- Regarding the secondary objectives, some that are described in the original published protocol will not be described in this SAP, but in SAPs for follow-up studies (see section 2.3).

2.2 Primary endpoint

The primary outcome is the rate of 'acute readmissions and ED visits' 12 months after discharge from the index hospital stay in the intervention group compared with the control group. An acute readmission is defined as any subsequent admission following the index admission excluding elective readmissions.

Specification:
- This is a composite endpoint combining "Acute readmissions" and "ED visits".
- We count all events per patient during 12 months from the index stay.

2.3 Secondary endpoints

Statistical analysis plan will be presented in this SAP only for the following secondary endpoints:

- Length of index hospital stay
- Time to first acute readmission after discharge from index hospital stay (12 months follow-up)
- The proportion of patients with acute readmissions within 30 days of discharge
- Mortality rate during 12 months' follow-up

Specification:<br>Some of the secondary endpoints from the protocol have been excluded from this SAP and will be presented in SAPs for follow-up studies.

3. Methods

3.1 General Study Design and Plan

We will recruit eligible participants to the study when they are admitted to the geriatric internal medicines ward (hereby called geriatric ward) in Tromsø or the general internal medicines ward (hereby called medicine ward) in Harstad. Participants will be randomized into two study arms, intervention and control (standard care) in a 1:1 relationship, stratifying on study site only. Randomization is performed after eligibility has been confirmed and patients have consented to participate. Consecutively, the intervention is commenced.

Study progress:

- The study started including patients in Tromsø on 22. September 2016
- The study started including patients in Harstad on March 2017
- Inclusion stopped in December 2019 in both study sites. The last patient was discharged from hospital on 22. December 2019
- Patients are followed-up for 12 months after discharge, and data will be collected after December 2020

3.2 Inclusion-Exclusion Criteria and General Study Population

Eligible patients were all patients aged ≥70 years admitted acutely to one of the study departments, independent of disease status, medication use, or whether they were able to consent.

Patients admitted to the intervention wards were included if they were willing to provide written informed consent during hospital stay (patient or next of kin).

Patients were excluded from the study if they met one of the following exclusion criteria:

- admitted to the study ward more than 72 hours before evaluation of eligibility
- moved to and discharged from other wards during the index stay
- unable to understand Norwegian (patient or next of kin)
- considered terminally ill or with a short life expectancy
- planned discharged on the inclusion day
- occupying a bed in a study ward but under the care of physicians from a non-study ward
- intervention from a study pharmacist considered necessary for ethical reasons (before randomization or in control group)

3.3 Randomisation and Blinding

Patients were randomized into intervention group and control group in a 1:1 relationship, only stratifying on study site. We applied block randomization with concealed and permuted randomization block sizes. The web-based randomization program was supplied by Unit for Applied Clinical Research, Faculty of Medicine Norwegian University of Science and Technology, Trondheim, which is an independent collaboration partner not involved in the project. Blinding was not feasible in this study, as everybody knew whether or not the intervention was delivered.

3.4 Sample Size

No data on hospitalization rates or visits to ED exist from our hospital. Therefore, data from a similar study in Sweden was applied as basis for sample size calculations. Gillespie et al. found a 16% reduction in visits to the hospital. In 12 months, patients in the intervention group had on average 1.5 visits and the intervention group 1.7 visits (2). If we expect a rate of 1.7 acute hospital visits per year in our control group, we would need a total of 456 patients to show a 16% reduction in hospital visits with a significance level of 5% and a power of 80% (Poisson regression). To compensate for dropout, we aimed to include 250 patients in each study group.

4 General Considerations

4.1 Timing of Analyses

The analyses of the endpoints specified in section 2.3 will be performed when 12 months follow-up data for all patients have been received, anticipated during May-June 2021.

4.2 Analysis Populations

Full Analysis Population: All patients included in the study and not withdrawing their consent, regardless of whether they were excluded after randomization.
Per Protocol Population: The full analysis population, except those who were excluded after randomisation.
Assigning patients to full analysis and per protocol population will be conducted before data on the primary endpoint is collected.

4.3 Variables, data sources and subgroups

Variables will be collected at the following time points during the study:
1) at baseline (during index hospitalization)
2) after follow-up from the following national registries:
- Norwegian Patient Registry (NPR) (hospitalizations and ED visits)
- The Norwegian Health Economics Administration Registry (ED visits)
- The National Cause of Death Registry (time and cause of deaths)

See Appendix 1 for details on the variables.
Variables that may influence on the primary endpoint will be investigated for interactions.

Subgroups
The following subgroups will be analysed for different treatment effects:
1. Number of medications at admission and discharge
2. Age groups 70–79, 80–89 and 90+
3. Patient responsibility for own medication management at discharge
4. Number and types of comorbidities at discharge
5. Number of hospital visits prior to inclusion

6. Length of index hospital stay
7. Referral from home, homecare or nursing home
8. Able to self-provide informed consent
9. Differences between study sites

Specification: We have added one subgroup (No 9 differences between study sites) which originally was not described in the published protocol.

4.4 Missing Data

We do not expect missing data for the primary endpoint as our national health registries are complete. When data is missing in independent variables, results will be presented with specified total number of patients contributing to each variable. In addition, for dependent and independent variables with more than 5% missing data, multiple imputation will be considered. Results of raw and imputed data will be presented.

4.5 Multi-centre Studies

The study sites in Tromsø and Harstad will be analysed together, but a subgroup analysis will be performed to investigate a possible centre effect. Regarding the intervention, the procedures, guidelines and working tools have been similar in both study sites and patients in the two study sites have been treated similarly. The only exception is with regard to collection of health-related quality of life. For this variable at baseline, all patients at study site Harstad were interviewed over telephone while for the Tromsø population, the first measurement was performed face-to-face.

4.6 Multiple Testing

To account for multiplicity, we will perform confirmatory significance testing for primary and secondary outcomes. All other significant tests will be treated as hypothesis generating. As we have conducted an RCT, we assume that any difference in baseline data is introduced by chance.

5 Summary of Study Data

5.1 Patient flow

The CONSORT flow diagram for the Patient flow will be developed, see Figure 5.1 below for a draft. Numbers that remain to be established: 1) Patients admitted to the hospital wards during study period, 2) Patients meeting the inclusion criteria, 3) Patients excluded and reasons why, 4) Patients dying during hospitalisation, 5) Patients dying during follow-up, 6) Patients lost to follow-up, 7) Patients in intention-to-treat analysis (ITT).

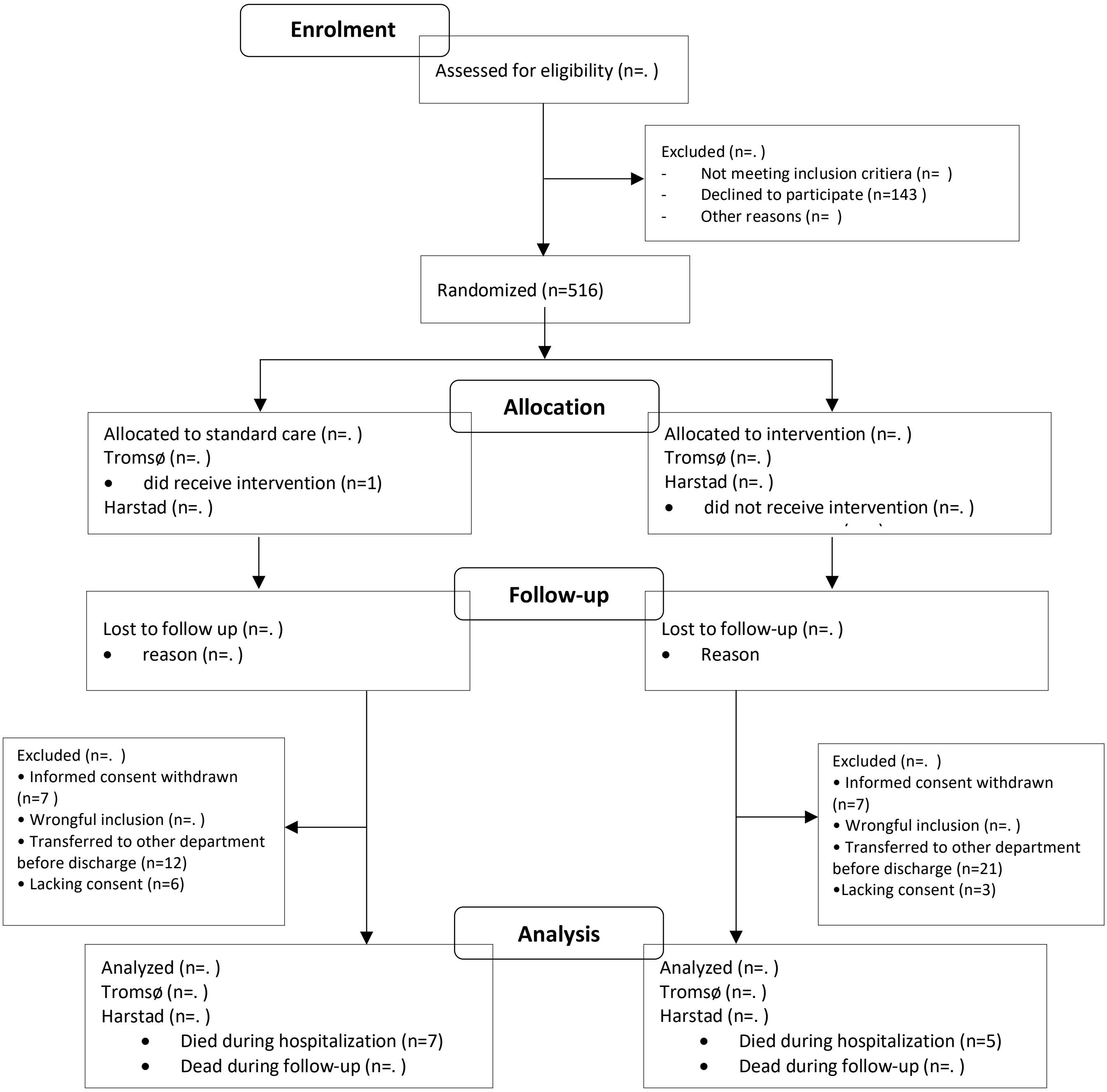

Figure 5.1 Participant flow diagram

5.2 Protocol deviations

Protocol deviations that we are aware of and could impact the analysis include:

- One patient randomised to control received intervention by a pharmacist for ethical reasons
- One patient randomized to intervention was wrongly excluded because due to discharge before the intervention could start

5.3 Demographic and Baseline Variables

See Table 5.1 for baseline characteristics collected for the study population. Most data, including photocopies/print of laboratory values, TILT (No; Tidlig Identifisering av Livstruende Tilstander, Eng; Early Identification of Life-threatening Conditions) form, medication chart and admission notes (including information on medical conditions), were collected before randomization to avoid information bias. MMS-score (mini-mental status), walking test results and health-related quality of life (HRQoL) measurements was collected after study inclusion.

Table 5.1: Baseline characteristics of study population (n=xxx)

	Intervention group n=xxx	Control group n=xxx
Age, mean years		
Sex, F, n (%)		
Study Site, n (%)		
Tromsø		
Harstad		
Ability to self-provide consent, n (%)		
Marital status, n (%)		
Married		
Divorced		
Single		
Educational level, n (%)		
Elementary school		
High school (mandatory)		
Higher education (<4 years)		
Higher education (>4 years)		
Admitted from, n (%)		
Home		
Nursing home		
Other hospital		
Living status, n (%)		
Home		
Nursing home		
Handling medications themselves, n (%)		
Yes		
No		
Assistance from municipality to handle medications, n (%)		
Yes		
No		
Receiving medications as multidose packages, n (%)		
Yes		
No		
Co-morbidity (Mean score CCI)		
Number of medications in use at hospital admission/discharge, n (%)		
Total		

Regular use	
Use as needed	
MMS-score (n=??) (mean score)	
Walking test results	
Health-related quality of life (EQ5D-VAS mean score)	

CCI; Charlson Comorbidity Index, EQ5D-VAS; EuroQoL 5L - health-related quality of life, MMS; mini-mental status

5.4 Concurrent Illnesses and Medical Conditions

Comorbidities will be described applying the Charlson Comorbidity Index and potentially other comorbidity scores, e.g. Rx-Risk comorbidity index (3, 4). Comorbidities defined during hospital admission will be collected from admission and discharge notes. Information about ICD codes will also be achieved from the national registry (NPR) to ensure completeness in comorbidities.

5.5 Prior and Concurrent Medications

- Medications at admission is defined by information in the hospital admission letter and first medication chart provided
- Medications at discharge is defined by information in the hospital discharge letter

5.6 Treatment Compliance (intervention fidelity)

In our study, treatment compliance will be defined by describing which part of the intervention was performed by the pharmacists for which patient. We will also analyse the medication-related problems identified by the pharmacists, their recommendations and physician agreement and the recommendations. This is prospectively denoted in the study database.

6 Efficacy Analyses

6.1 General on statistics

We will investigate data for normality and apply the appropriate statistical tests. A two-sided 5% significance level will be applied, with no adjustments for multiplicity.

All analyses will be performed applying SPSS for windows or Mac. P-values < 0.05 will be regarded as statistically significant. P-values ≥0.001 will be reported to 3 decimal places; p-values less than 0.001 will be reported as "<0.001". The mean, standard deviation, and any other statistics other than quantiles, will be reported to one decimal place greater than the original data. Quantiles, such as median, or minimum and maximum will use the same number of decimal places as the original data. Estimated parameters, not on the same scale as raw observations (e.g. regression coefficients) will be reported to 3 significant figures.

6.2 Intention to treat and per protocol analysis

The main analysis will be performed according to the intention to treat (ITT) principle. In the ITT analysis all patients are analysed according to their initially assigned study arm at baseline, regardless of adherence to study protocol. Patients who withdrew consent or patients with a protocol violation concerning eligibility are excluded from the ITT analysis. Patients lost to follow-up will likewise be excluded from the ITT analysis. Per protocol analysis will also be performed. All subjects from the ITT population without protocol violations and deviations regarding treatment will be included in the PP population. See Figure 6.1 for illustration of ITT and PP analyses.

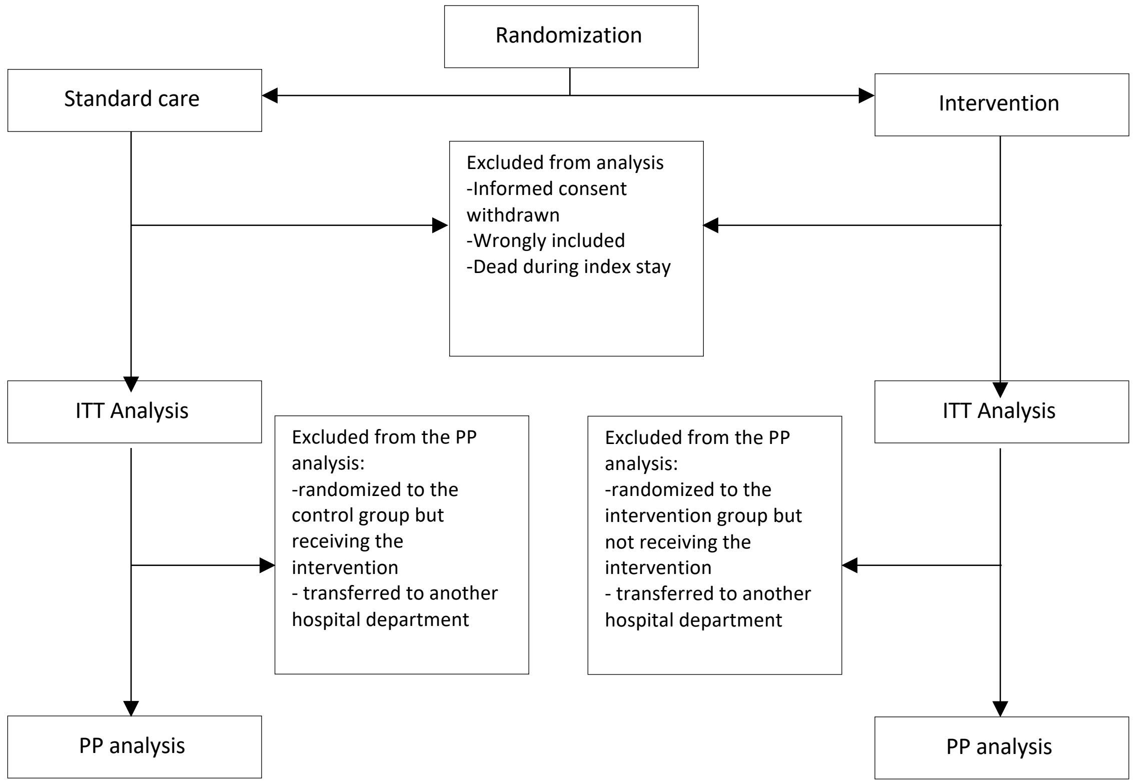

Figure 6.1: Patient inclusion for intention-to-treat (ITT) and per protocol (PP) analysis

6.3 Primary endpoint analysis

The composite endpoint

The primary endpoint is defined as the composite endpoint “acute readmission and ED visits” 12 months after discharge from the index hospital stay in the intervention group compared with the control group. Consequently, the endpoint comprises both “readmissions” and “visit to emergency departments (ED)”.

Hypothesis

- The H0 hypothesis is: The intervention does not influence the number of primary endpoints during 12 months after index hospitalization.
- The H1 hypothesis is: The intervention influences the number of primary endpoints during 12 months after index hospitalization.

Figure 6.2 illustrates how we assume that the endpoint will occur during the follow-up time.

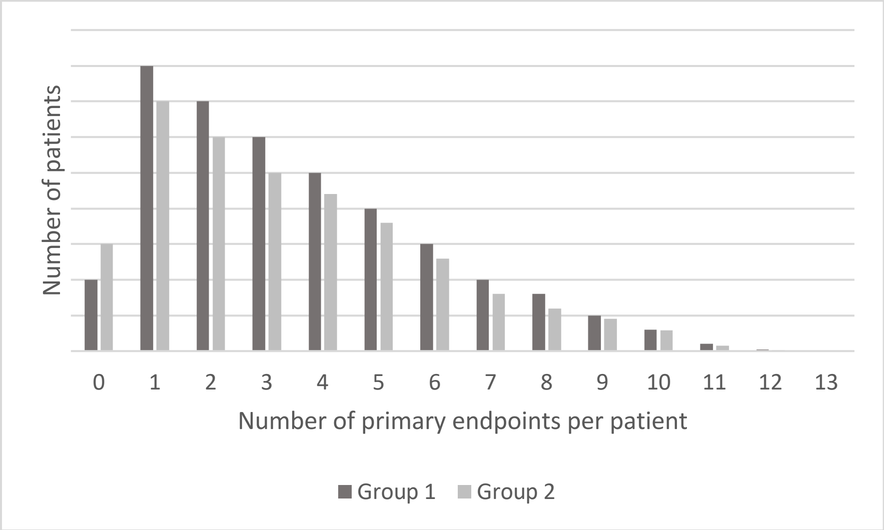

a)

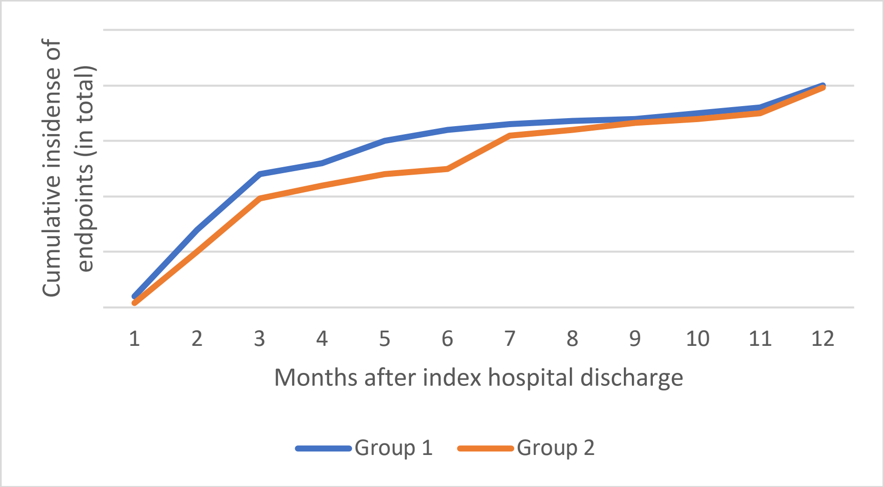

b)

Figure 6.2: Illustrations of primary endpoints during a 12-month follow-up time for patients in the two groups, assuming that we will see a difference between the groups.
a) Number of patients having 1-x number of endpoints during the 12-month follow-up period.
b) Cumulative number of endpoints (in total) 1-12 months after index hospital discharge.

Person-time (time under risk for experiencing an endpoint) contribution
Each patient may experience many endpoints during the 12-month follow-up, and the primary endpoint per patient will be related to the patient´s person-time contribution during the 12-month follow-up period after index hospital discharge.

To account for that a new endpoint cannot occur in the period a patient is “in an endpoint” (i.e. time in hospital if patient is hospitalised), and that patients may die before the end of the 12-month follow-up period, we will calculate person-time contribution for all patients, which is “time outside hospital” in the follow-up period where the person is still alive. Consequently, total person time contribution per patient =
365 days follow-up time after hospital discharge, minus “time in an endpoint” minus time after (potential) death.

Primary endpoint analysis

Specification
According to our published protocol, the primary analysis will be a Poisson regression comparing the rate of the composite endpoint during 12 months after discharge between the two study groups. In this SAP, we specify that the primary endpoint will be investigated by comparing the rate of events (also recurring) happening in the intervention group and the control group during the 12-month follow-up period, taking into account the specific person-time contribution per patient (when the patients are at risk for experiencing an endpoint).<br><br>We will supplementarily perform a Poisson regression analysis if we need to adjust for crucial differences between the study groups. This we will not know before we have the data in house.

6.4 Secondary endpoint analysis

The secondary endpoints to be analysed is presented in Table 6.1 together with the outcome measure and methodology.

Table 6.1: Overview of outcome measures and methods of analysis to investigate secondary endpoints.

Variable/outcome		Outcome measure	Methods of analysis
1)	Length of hospitalisation (LOS) during index stay	Days [continuous]	T-test, and potentially Anova
2)	Time to first unplanned readmission within 12 months after discharge from index hospital stay	Days [continuous]	Kaplan Meier method and the log-rank test. Hazard ratios (HRs) with 95% confidence intervals will be estimated using a Cox proportional hazards model.
3)	The proportion of patients readmitted acutely within 30 days	Proportion	Xhi-square test
4)	12-month mortality	Proportion	Total mortality will be analysed as a time-to-event analysis. The Kaplan Meier method and the log-rank test will be applied. Further, a Cox proportional HR model will be applied to estimate HRs. HRs will be presented with 95% confidence intervals.

6.5 Blinding

The dataset will be prepared for analysis by the project administers who are familiar with the study and the variables (JSJ, KHH and BHG).

The main analyses will be performed by a statistician (FS) blinded for group allocation and not involved in data collection, data punching or in preparing the data files for analyses. To maintain blinding and prevent bias, data analyses on the primary endpoint will be performed as follows: JSJ/BHG/KHH prepare a data file including a new variable indicating whether patients are in the intervention group or in the control group. This new variable is blinded, and allocation code is stored safely and not provided to the statistician (FS). FS receives the dataset with the new code for the allocated group and carry out the primary analysis. When the statistical analysis is finalized, group allocation will be revealed by JJSH/BHG/KHH with FS present.

7 Summary of Changes to the Protocol

Compared to the published protocol and the information denoted in www.clinicaltrials.gov, the following amendments have now been made in the SAP:

1) The calculation of a retrospective Charlston Comorbidity index on all participants and the potential use of this as a covariate in analysis.
2) In the subgroup analysis overview, we have added a comparison of outcomes of patients from the two study sites Tromsø and Harstad and the number and type of comorbidities at discharge.
3) The primary endpoint analysis may not necessarily be a Poisson Regression analysis, but a comparison of rates of endpoints experienced by intervention group patients vs. control group patients.

8 References

1. Johansen JS, Havnes K, Halvorsen KH, Haustreis S, Skaue LW, Kamycheva E, et al. Interdisciplinary collaboration across secondary and primary care to improve medication safety in the elderly (IMMENSE study): study protocol for a randomised controlled trial. BMJ Open. 2018;8(1):e020106.

2. Gillespie U, Alassaad A, Henrohn D, Garmo H, Hammarlund-Udenaes M, Toss H, et al. A comprehensive pharmacist intervention to reduce morbidity in patients 80 years or older: a randomized controlled trial. Arch Intern Med. 2009;169(9):894-900.

3. Charlson Comorbidity Index (CCI) [cited 2020 20.04]. Available from: https://www.mdcalc.com/charlson-comorbidity-index-cci.

4. Pratt NL, Kerr M, Barratt JD, Kemp-Casey A, Kalisch Ellett LM, Ramsay E, et al. The validity of the Rx-Risk Comorbidity Index using medicines mapped to the Anatomical Therapeutic Chemical (ATC) Classification System. BMJ Open. 2018;8(4):e021122.

Appendix 1: Variables collected for use in primary and secondary analyses

Table A1: Variables that are and will be collected for the study participants.

Variable	Variable type	Definitions	Data source
Length of index hospital stay (actual)	Continuous	The number of days the patient was admitted to hospital during the index stay. NB! The patient may have been ready for discharge earlier, but because of no space in municipality he/she could not be sent out. These days are denoted and will be subtracted from the number shown in the patient registry.	National registry<br>Study database*
Length of index hospital stay (when ready to be discharged)	Continuous	The number of days the patient was admitted to hospital during the index stay minus the number of days the patient was hospitalized after he/she was ready for discharge. NB! The patient may have been ready for discharge earlier, but because of no space in municipality he/she could not be sent out. These days are denoted and will be subtracted from the number shown in the patient registry.	National registry<br>Study database*
Number of unplanned hospital admissions in the year preceding the index stay	Continuous	12 months' follow-up, 6 months before and after data for adjusting for secular trends	National registry
Number of deaths in the year preceding the index stay	Continuous	12 months' follow-up, 6 months before and after data for adjusting for secular trends	National registry
Number of unplanned visits to ED departments in the year preceding the index stay	Continuous	12 months' follow-up, 6 months before and after data for adjusting for secular trends	National registry
Living status	Categorical	1.home with home-care,<br>3. nursing home permanent living,<br>4. nursing home short term	Study database*
Responsible for administering their own medication on admission to index stay	Categorical	Yes<br>No<br>Partial	Study database*
Receiving multidose packed drugs at admission to index hospital stay	Categorical	Yes<br>No	Study database*
Medications in regular use and use as needed at admission and discharge	Continuous	Name of medications (ATC level 5) in regular use exluding pro re nata drugs	Study database*
Charlson comorbidity index score at admission/discharge	Continuous	Score	Based on data in study database
Age	Continuous and Categorical	Years<br>70–79<br>80–89<br>90+	Study database*<br>& from national registry
Sex	Categorical	Male 2. female	Study database*<br>& from national registry
Study site	Categorical	Tromsø<br>Harstad	Study database* & from randomization database
Able to self-provide informed consent or not.	Categorical	Yes<br>No	Study database*

Educational status	Categorical	1. Grunnskole<br>2.frammansskole eller folkehøyskole<br>3. Yrkesfaglig videregående, Realskole, eller yrkesskole<br>4.Allmennfaglig videregående skole eller gymnas<br>5. Høyskole eller universitet under 4 år<br>6. Høyskole eller universitet over 4 år	Study database*
Receiving help from PSHT (patient centered health care team) at admission or discharge.	Categorical	Yes, No	Study database*
Kidney function (eGFR) at admissjon	Continuous	First value from index hospitalization.	Study database*
Help from the municipality	Continuous	Number of hours per week that the patient receives of home care services from the municipality	Collected from the municipalities at 1, 6 and 12 months after discharge.
Health-relate Quality of life	Continuous	EQ5D-VAS scale	Collected at baseline, at 1, 6 and 12 months.

* Data will be collected prospectively from patient journal and pharmacist work during the study period and denoted in a de-identified study database where patients are given a study ID number. The study database includes both intervention and control patients, and a code list is kept separate
