# Supplementary material for: Interdisciplinary collaboration across secondary and primary care to improve medication safety in the elderly (The IMMENSE study) – a randomized controlled trial
Source: BMC Health Serv Res. 2022 Oct 26;22:1290. doi: 10.1186/s12913-022-08648-1 (PMC9597977; doi:10.1186/s12913-022-08648-1)
Supplement: Supplementary file 2 — Additional file 2. [file 12913_2022_8648_MOESM2_ESM.docx]

Supplementary material 2

sTable 1 Baseline characteristics in the per-protocol population (n = 442)

|  | | Intervention group  (n=221) | | | Control group  (n=221) | |
| --- | --- | --- | --- | --- | --- | --- |
| Age, mean years (SD) | | 83.3 | | (6.4) | 83.0 | (6.3) |
| Sex, female, n (%) | | 140 | | (63.3) | 118 | (53.4) |
| Study Site, n (%) | |  | |  |  |  |
|  | Geriatric ward (study site 1) | 181 | | (81.9) | 182 | (82.4) |
|  | General medicine ward (study site 2) | 40 | | (18.1) | 39 | (17.6) |
| Ability to self-provide consent, n (%) | | 158 | | (71.5) | 149 | (67.4) |
| Marital status, n (%) | |  | |  |  |  |
|  | Widow/widower | 99 | | (44.8) | 94 | (42.5) |
|  | Married/live in partner | 86 | | (38.9) | 85 | (38.5) |
|  | Single | 23 | | (10.4) | 25 | (11.3) |
|  | Divorced/separated | 11 | | (5.0) | 14 | (6.3) |
|  | Missing | 2 | | (1.0) | 3 | (1.4) |
| Educational level, ISCED level n (%) | | | | |  |  |
|  | Elementary school, level 1 | 102 | | (46.2) | 106 | (48.0) |
|  | Secondary education, level 2-3 | 79 | | (35.7) | 74 | (33.5) |
|  | Higher education (<4 years), level 5-6 | 21 | | (9.5) | 17 | (7.7) |
|  | Higher education (>4 years), level 7-9 | 11 | | (5.0) | 10 | (4.5) |
|  | Missing | 8 | | (3.6) | 14 | (6.3) |
| Living status at admission, n (%) | |  | |  |  |  |
|  | Home, no help from home care services | 78 | | (35.3) | 62 | (28.1) |
|  | Home, with help from home care services | 107 | | (48.4) | 133 | (60.2) |
|  | Nursing home, short term | 19 | | (8.6) | 11 | (5.0) |
|  | Nursing home, permanent | 17 | | (7.7) | 15 | (6.8) |
| Discharge to home, n (%) | | 136 | | (61.5) | 126 | (57.0) |
| Handling medications themselves, n (%) | |  | |  |  |  |
|  | Yes | 83 | | (37.6) | 71 | (32.1) |
|  | No | 96 | | (43.4) | 98 | (44.3) |
|  | Partly | 42 | | (19.0) | 52 | (23.5) |
|  | Missing |  | |  |  |  |
| Co-morbidity^b^ (Median score,IQR) | |  | |  |  |  |
| Charlson comorbidity index | | 2 | | (1-3) | 2 | (1-4) |
| Number of medications (ATC-codes) in use at hospital admission, Median (IQR) | | |  | | | |
|  | Total | 8 | | (5-12) | 9 | (6-13) |
|  | Regular use | 6 | | (4-9) | 7 | (4-10) |
|  | Use as needed | 2 | | (0-3) | 2 | (0-3) |
| Medical history in admission notes, n (%) | |  | |  |  |  |
|  | Hypertension | 44 | | (19.9) | 48 | (21.7) |
|  | Atrial fibrillation | 112 | | (50.7) | 105 | (47.5) |
|  | Asthma or COPD | 63 | | (28.5) | 62 | (28.1) |
|  | Diabetes Mellitus | 51 | | (23.1) | 51 | (23.1) |
|  | Heart failure | 35 | | (15.8) | 34 | (15.4) |
|  | Dementia | 31 | | (14.0) | 32 | (14.5) |
| Emergency medical visits one year before index hospital stay | | | | | | |
| Emergency medical visits, n (% with one) | | 414 | | (67.9) | 517 | (72.4) |
| Emergency medical visits, median (IQR) | | 1 | | (0-3) | 1 | (0-3) |

ATC; anatomical therapeutic chemical classification system, ; F; female, IRQ: interquartile range, ISCED; international standard classification of education, SD; standard deviation. a) educational level categorized by the international standard classification of education b) Co-morbidity based on diagnosis in admission and discharge papers.

sTable 2 Effect of the intervention on the primary endpoint (Rate of emergency medical visits one year after discharge) in the different subgroups of the ITT-population

| Subgroup | | Number of patients in subgroup | Intervention | Control | Incidence rate ratio (95 % CI) Intervention compared with control | |
| --- | --- | --- | --- | --- | --- | --- |
|  | | (intervention/  control) | Number of events | Number of events | Crude | Adjusted^a^ |
| Number of medications at admission | | | |  |  |  |
|  | 0-5 | 120 (68/52) | 104 | 68 | 1.09 (0.62-1.94) | 1.10 (0.63-1.92) |
|  | 6-10 | 185 (97/88) | 211 | 167 | 1.26 (0.90-1.78) | 1.36 (0.97-1.89) |
|  | >10 | 175 (79/96) | 182 | 264 | 0.77 (0.55-1.09) | 0.79 (0.59-1.08) |
| Age at inclusion, years | | | |  |  |  |
|  | 70-79 | 139 (69/70) | 136 | 140 | 1.07 (0.66-1.73) | 1.01 (0.65-1.56) |
|  | 80-89 | 264 (136/128) | 268 | 293 | 0.93 (0.69-1.26) | 1.05 (0.79-1.38) |
|  | ≥90 | 77 (39/38) | 93 | 66 | 0.84 (0.50-1.40) | 0.95 (0.58-1.57) |
| Responsible for own medication at discharge, n | | | |  |  |  |
|  | yes | 108 (57/51) | 83 | 103 | 0.73 (0.46-1.15) | 0.80 (0.51-1.26) |
|  | no | 283 (142/141) | 257 | 254 | 0.98 (0.72-1.33) | 1.09 (0.82-1.46) |
|  | Partially | 81 (43/38) | 144 | 134 | 1.02 (0.64-1.62) | 1.01 (0.68-1.50) |
|  | missing | 8 |  |  | - | - |
| Comorbidity, Charlson Comorbidity Index | | | |  |  |  |
|  | 0-2 | 262 (140/122) | 266 | 217 | 1.05 (0.77-1.43) | 1.07 (0.81-1.42) |
|  | >2 | 218 (104/114) | 231 | 282 | 0.91 (0.65-1.26) | 1.01 (0.74-1.39) |
| Emergency medical visits in the year before index stay, n | | | | |  |  |
|  | 0-1 | 252 (132/120) | 210 | 169 | 1.05 (0.75-1.47) | 1.05 (0.75-1.47) |
|  | >1 | 228 (112/116) | 287 | 330 | 0.92 (0.69-1.23) | 0.98 (0.75-1.29) |
| Length of index hospital stay, days | | | |  |  |  |
|  | 0-6 | 270 (145/125) | 301 | 248 | 0.99 (0.74-1.34) | 1.01 (0.77-1.32) |
|  | >6 | 210 (99/111) | 196 | 251 | 0.93 (0.64-1.34) | 1.07 (0.75-1.51) |
| Admitted from, n | |  |  |  |  |  |
|  | Home, no help from municipality | 157 (88/69) | 127 | 130 | 0.83 (0.55-1.24) | 0.86 (0.59-1.26) |
|  | Home, with help from municipality | 255 (116/139) | 317 | 310 | 1.18 (0.88-1.59) | 1.26 (0.96-1.65) |
|  | Nursing home | 68 (40/28) | 53 | 59 | 0.61 (0.30-1.24) | 0.64 (0.32-1.28) |
| Ability to consent, n | |  |  |  |  |  |
|  | Yes | 334 (174/160) | 394 | 371 | 1.04 (0.79-1.36) | 1.07 (0.84-1.36) |
|  | No | 146 (70/76) | 103 | 128 | 0.74 (0.47-1.67) | 0.85 (0.55-1.33) |
| Study site, n | |  |  |  |  |  |
|  | Geriatric ward | 389 (198/191) | 411 | 402 | 0.97 (0.74-1.25) | 1.02 (0.81-1.30) |
|  | General medicine ward | 91 (46/45) | 86 | 97 | 0.88 (0.52-1.51) | 1.07 (0.65-1.78) |

1. Adjusted for the number of emergency medical visits in the year before index hopitalization.

sTable 3 Primary and secondary outcomes in the per-protocol population (n=442)

| Primary outcome after 12 months | | Intervention | | Control | |  | |
| --- | --- | --- | --- | --- | --- | --- | --- |
|  |  | (n=221) | | (n=221) | | Crude | Adjusted^a^ |
|  | | n, median (IQR) | | n, median (IQR) | | Incidence rate ratio (95 % CI) | |
| Emergency medical visits | | 434 | 1 (0-3) | 472 | 1 (0-3) | 0.90 (0.70-1.14) | 0.97 (0.77-1.21) |
|  | ED-visits | 245 | 1 (0-2) | 263 | 1 (0-2) | 0.89 (0.68-1.18) | 0.94 (0.72-1.23) |
|  | Rehospitalisation | 189 | 1 (0-1) | 209 | 0 (0-1.5) | 0.90 (0.67-1.21) | 0.97 (0.74-1.27) |
| Secondary outcomes | |  | |  | |  | |
| Days to first event | | median (%) | | median (%) | | Hazard rate (95 % CI) | |
|  | Readmission | 329 | (50.7) | 351 | (47.5) | 1.01 (0.78-1.32) | 1.07 (0.82-1.40) |
|  | Emergency medical visit | 143 | (70.6) | 108 | (71.0) | 0.89 (0.71-1.11) | 0.92 (0.74-1.15) |
|  | | n (%) | | n (%) | | Odds ratio (95 % CI) | |
| Readmissions within 30 days | | 23 | (10.4) | 32 | (14.5) | 0.69 (0.39-1.22) | 0.78 (0.43-1.41) |
| All-cause mortality within 12 months | | 40 | (18.1) | 45 | (20.4) | 0.86 (0.54-1.39) | 0.90 (0.56-1.46) |

IQR; Interquartile Range a) Adjusted for the number of emergency medical visits during 365 days prior to the index hospital stay.^a)^Adjusted for study site and the number of emergency medical visits one year before index hospital stay.
